# Can you feel the force just right? Tactile force feedback for training of minimally invasive surgery—evaluation of vibration feedback for adequate force application

**DOI:** 10.1007/s00464-024-10919-3

**Published:** 2024-06-04

**Authors:** Felix von Bechtolsheim, Florian Bielert, Sofia Schmidt, Nathalie Buck, Sebastian Bodenstedt, Stefanie Speidel, Lisa-Marie Lüneburg, Thomas Müller, Yichen Fan, Tina Bobbe, Luca Oppici, Jens Krzywinski, Jakob Dobroschke, Jürgen Weitz, Marius Distler, Florian Oehme

**Affiliations:** 1 Department of Visceral, Thoracic, and Vascular Surgery, Faculty of Medicine and University Hospital Carl Gustav Carus, TUD Dresden University of Technology, Fetscherstraße 74, 01307 Dresden, Germany; 2 Division of Translational Surgical Oncology, National Center for Tumor Diseases Dresden, Dresden, Germany; 3 Industrial Design Engineering, Technische Universität Dresden, Dresden, Germany; 4 Centre for Tactile Internet With Human-in-the-Loop (CeTI), Technische Universität Dresden, Dresden, Germany; 5 Department of Teacher Education and Outdoor Studies, Norwegian School of Sport Sciences, Oslo, Norway; 6 Department of General, Visceral and Thoracic Surgery, Proctology, Helios Klinikum Pirna, Pirna, Germany

**Keywords:** Tactile feedback, Force feedback, Vibration, Minimally invasive surgery, Training

## Abstract

**Background:**

Tissue handling is a crucial skill for surgeons and is challenging to learn. The aim of this study was to develop laparoscopic instruments with different integrated tactile vibration feedback by varying different tactile modalities and assess its effect on tissue handling skills.

**Methods:**

Standard laparoscopic instruments were equipped with a vibration effector, which was controlled by a microcomputer attached to a force sensor platform. One of three different vibration feedbacks (F1: double vibration > 2 N; F2: increasing vibration relative to force; F3: one vibration > 1.5 N and double vibration > 2 N) was applied to the instruments. In this multicenter crossover trial, surgical novices and expert surgeons performed two laparoscopic tasks (Peg transfer, laparoscopic suture, and knot) each with all the three vibration feedback modalities and once without any feedback, in a randomized order. The primary endpoint was force exertion.

**Results:**

A total of 57 subjects (15 surgeons, 42 surgical novices) were included in the trial. In the Peg transfer task, there were no differences between the tactile feedback modalities in terms of force application. However, in subgroup analysis, the use of F2 resulted in a significantly lower mean-force application (*p*-value = 0.02) among the student group. In the laparoscopic suture and knot task, all participants exerted significantly lower mean and peak forces using F2 (*p*-value < 0.01). These findings remained significant after subgroup analysis for both, the student and surgeon groups individually. The condition without tactile feedback led to the highest mean and peak force exertion compared to the three other feedback modalities.

**Conclusion:**

Continuous tactile vibration feedback decreases the mean and peak force applied during laparoscopic training tasks. This effect is more pronounced in demanding tasks such as laparoscopic suturing and knot tying and might be more beneficial for students. Laparoscopic tasks without feedback lead to increased force application.

**Supplementary Information:**

The online version contains supplementary material available at 10.1007/s00464-024-10919-3.

Minimally invasive surgery has become a standard approach in all surgical fields, with laparoscopy representing the most common procedure. However, the basic functionality of most laparoscopic instruments has remained largely unchanged since the invention and widespread implementation of laparoscopic surgery almost four decades ago. The lack of improvement in laparoscopic instruments has contributed to the persistence of major disadvantages associated with laparoscopy, namely, poor ergonomic and reduced haptic feedback response to laparoscopic instruments [1–3].

The importance of well-designed laparoscopic instruments is underlined by the results of a survey by Alleblas et al. in which 77% of surgeons reported physical complaints related to the use of standard laparoscopic instruments. Additionally, the participants of this survey saw an added value of instruments with improved haptic feedback in feeling, for example, different types of tissue or an arterial pulse [1].

However, haptic feedback has significant added value, especially for surgical novices, when training for minimally invasive surgery [4, 5]. In fact, Zhou et al. observed that participants in a training course showed more consistent performance and a steeper learning curve when using simulators with haptic feedback than when training on simulators without haptic feedback [6]. Even manual exploration of an organ has been shown to enhance subsequent laparoscopic tissue manipulation of the same organ, as demonstrated by Postema et al. [7].

This observation is not surprising considering that the sensitivity of laparoscopic instruments is impaired by a factor of 8–20 compared to the sensitivity of human hands [8]. This loss in sensitivity is mainly caused by friction between the instrument and the trocar, which corrupts the discrimination of forces resulting from instrument tissue interactions and frictional forces [8, 9].

Nevertheless, the haptic feedback of human hands seems unmatched, even if laparoscopic instruments are equipped with tactile sensors. However, the method of delivering feedback might also be relevant, as there is evidence that visual feedback of force application might be disadvantageous compared to haptic feedback [10].

Consequently, the integration of haptic feedback could help improve tissue handling and associated training. Therefore, the aim of this study was to investigate the influence of tactile feedback, which makes up a significant proportion of haptic feedback but also enables the perception of force via vibration, for example, on the application of force during laparoscopic exercises and to compare several tactile feedback modalities. Moreover, understanding the influence of tactile feedback on the overall user experience is essential. Suboptimal system responses, characterized by delays or sluggishness, may negatively affect perceived effectiveness, leading to impatience, frustration, or even anger. In contrast, a forward-thinking and innovative design can influence perceived aesthetics, eliciting emotions, such as surprise, curiosity, or pleasure. Ultimately, all three components of user experience—functionality, aesthetics, and emotional response—contribute to the overall appraisal of the system, influencing the user’s future decisions and behavior [11]. Therefore, a comprehensive evaluation of tactile feedback in laparoscopic devices is essential for optimizing both technical and user-related aspects of the system.

To our knowledge, the comprehensive tactile experience in the context of laparoscopic surgery training has not been explored. Therefore, the aim of this work to investigate the tactile experience by addressing the following research question: How do participants emotionally react to tactile feedback and how is it interpreted?

## Methods

This trial was designed and conducted as a prospective crossover multicenter study with a randomized order of different tactile feedback applications. The trial protocol was approved by the ethics committee of TU Dresden (decision number EK 285072016). The experiments were carried out according to the relevant guidelines. The draft and writing of this article were aligned with the CONSORT statement [12].

### Experimental setup

**Fig. 1:**
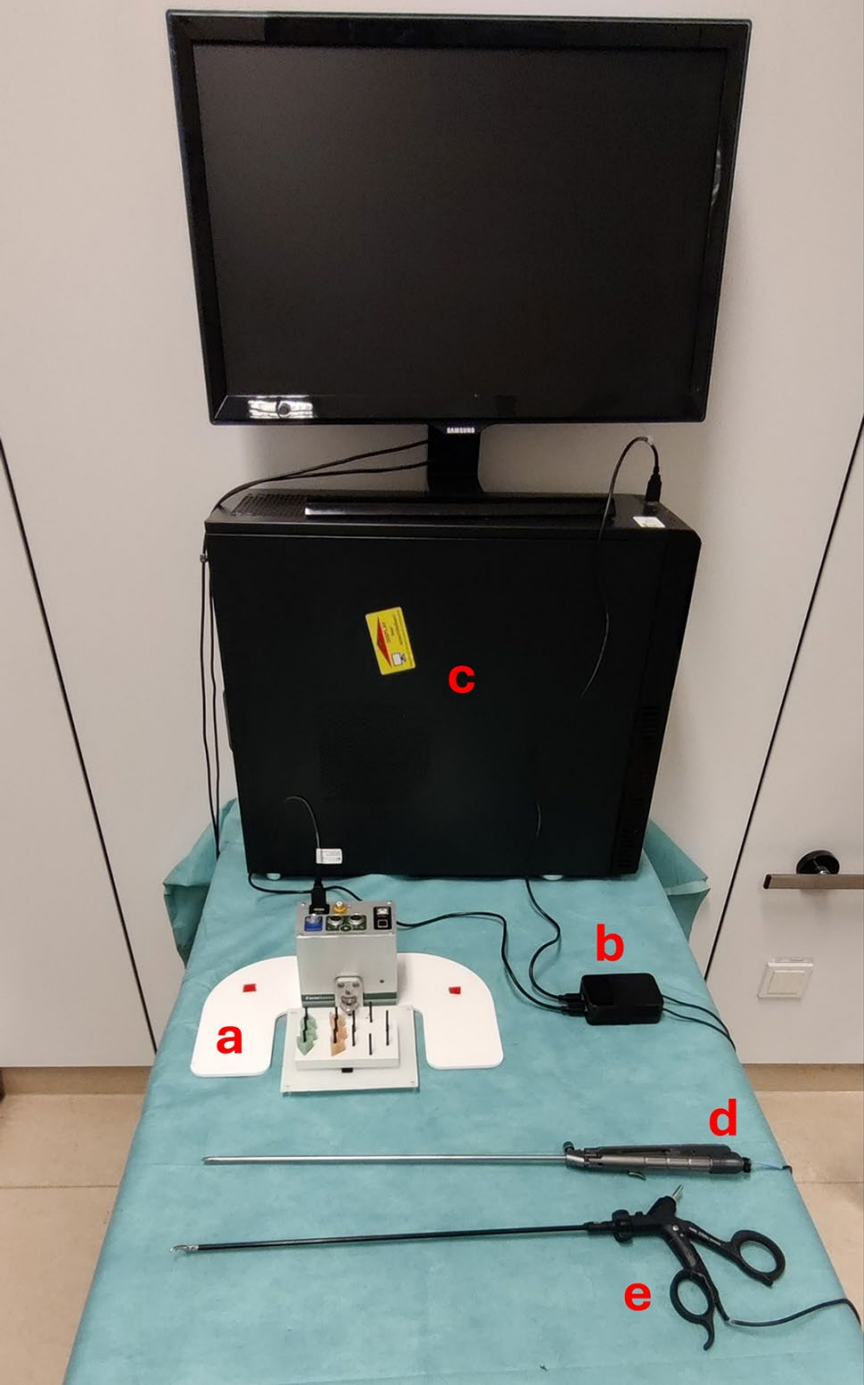
Experimental setup with **a** force measuring sensor (ForceTrap®, Fa. Medishield, The Netherlands), connected to a **b** Arduino Microcontroller, which was connected to the **c** workstation and controlled the vibration effectors attached to the **d** laparoscopic needle holder and the **e** laparoscopic grasper (not shown is the laparoscopic box trainer)

For this trial, a special experimental setup was developed using a force sensor (ForceTrap® by MediShield) connected to a workstation (Ubuntu 18.04), which continuously captured the force data in three dimensions (Fig. 1). An Arduino-based microcontroller connected to the workstation controlled two small vibration effectors attached to the handle of a laparoscopic needle holder (Storz, Tuttlingen, Germany), a laparoscopic grasper (Storz, Tuttlingen, Germany) and laparoscopic scissors (Pajunk, Geisingen, Germany). The force sensor was placed within a standard Fundamentals of Laparoscopic Surgery (FLS) box trainer (Limbs & Things, Savannah, Georgia, USA). The tasks were placed on platforms connected to the force sensor, which could measure all forces in three dimensions.

The force input was then processed by the PC using self-designed software. The program captured the sensor stream from the ForceTrap while thresholding the measured absolute force to determine if and what feedback to provide by triggering the microcontroller. The feedback modalities were designed with the help of a toolkit (HapticLabs, Dresden, Germany, https://www.hapticlabs.io/). The toolkit utilized made it possible for the first time to vary three tactile feedback parameters that characterize the actual vibration: relative intensity (0–100%), frequency (0–190 Hz), and duration (ms). The vibration effectors were unbalanced motors (model: Leader-3039).

A total of three different feedback modalities were designed based on the recommendations of a board consisting of two experienced surgeons and three technical designers. A fourth modality without any tactile feedback was included as a control (Supplementary Material).

*Feedback modality 1 (F1)*: Double vibration impulse if the applied force exceeds 2 N.

*Feedback modality 2 (F2)*: The vibration impulse increases linearly to the force, starting at a minimal force of 0.14 N and extending beyond 2 N, with a double vibration impulse at 2 N of force application.

*Feedback modality 3 (F3)*: Single vibration impulse at 1.5 N and double vibration impulse if force application exceeds 2 N.

*No feedback (F4)*: No feedback at all.

### Participants

Laparoscopic novices without experience in laparoscopic surgery and surgeons were included from two surgical centers (University Hospital Carl Gustav Carus Dresden and Helios Klinikum Pirna). Written consent was obtained from all participants. Before participation in the trial, all laparoscopic novices had to finish a standardized basic laparoscopic skills training course and reach a predefined level of proficiency [13]. Only surgeons with previous experience as first surgeon in laparoscopy were included. Basic participant characteristics (age, surgical and robotic experience, handedness, vision irregularities, etc.) were evaluated using a questionnaire.

### Study design

**Fig. 2:**
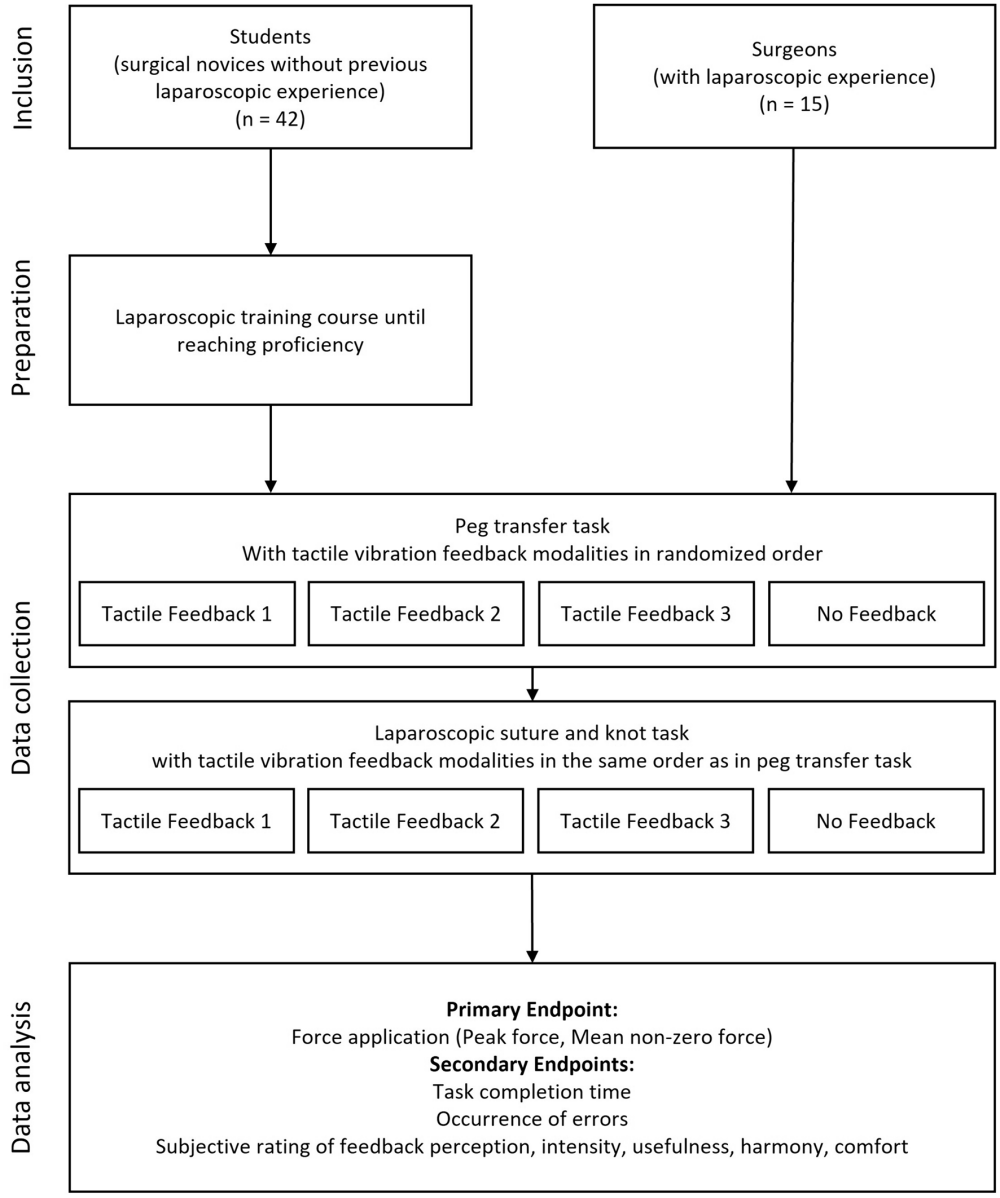
Trial scheme

The study was conducted at two locations (University Hospital Carl Gustav Carus Dresden and Helios Klinikum Pirna). After answering the questionnaire regarding basic participant characteristics, all participants performed two tasks in sets of four repetitions with all four feedback modalities in a randomized order (Fig. 2). First, a peg transfer task was performed a total of four times and then, participants performed a laparoscopic suture and knot task, again a total of four times. Here, the order of the feedback modalities remained the same as during the first set. After each repetition, participants were asked for their subjective evaluation of the respective feedback. The survey included questions about feedback perception, intensity, usefulness, harmony, and comfort.

### Variables

The primary endpoint was the force applied during task performance. The trials were performed on a platform attached to a force measuring device (ForceTrap®, MediShield B.V., Delft, The Netherlands), which measured all forces applied to the platform. The data was then uploaded and stored in the ForceSense NET database for later analysis. The variables of interest for the primary endpoint were as follows:

 − Peak force: the maximum force applied during any task [14].
 − Mean non-zero force: average force over the duration of a task excluding periods with zero force exertion [14].

The secondary endpoints were the time needed to complete a task and the occurrence of predefined errors (Supplementary material). There was one error criterion for Peg transfer (triangle lost) and three error criteria for the laparoscopic suture and knot task (imprecise suturing, insufficient approximation, and knot tightness). One investigator recorded the occurrence of these errors during any tasks.

### User experience and subjective evaluation of feedback modalities

To measure the user experience, a survey was used to cover pragmatic (e.g., efficiency or ergonomics) and hedonic (e.g., aesthetics, affect, or meaning) dimensions according to Hassenzahl [15]. Feedback 4 (no feedback) was excluded from the statistical analysis for the subjective evaluation of the different feedback modalities.

For pragmatic aspects, the overall perception (perception and intensity) was rated using a Likert scale from 1 to 5 and the perceived usefulness was rated with a Likert scale ranging from 1 to 4 (Supplementary material):

 − Perception (“How was the feedback perceived?”) was measured on a scale ranging from “I have not felt any feedback” to “The feedback was precise and very well perceptible.”
 − Intensity (“What did the feedback feel like?”) was measured on a scale ranging from “very weak” to “very strong.”
 − Perceived usefulness (“How useful would you consider this feedback in fulfilling the tasks?”) was measured on a scale ranging from “not useful” to “very useful.”

To assess how participants experienced tactile feedback on a hedonic level, the two most relevant dimensions from Kim and Schneider were included in the questionnaire: Harmony (“How well does the feedback fit into the task situation?”) and Autotelics/Comfort (“How does the feedback feel?”) [16]. Participants indicated their response using a 100-point Likert scale, choosing any value between 0 and 100 (0 = no feedback, 1 = very bad/uncomfortable; 100 = very good/comfortable).

### Statistical analysis

Data analysis and statistical analysis were performed using SPSS version 28 (IBM Corp, Armonk NY, USA). The frequency distribution was inspected, and the Kolmogorov–Smirnov test was used to test the normality of continuous data. The data are represented either as the distribution of frequencies or as the mean values (mean) with standard deviation (SD) for continuous variables. For analysis of differences between the various feedback modalities, a general linear model for repeated measures with post hoc Bonferroni correction was used. For the analysis of differences in error occurrence between the various feedback modalities, a Cochran’s *Q* test for related samples was chosen. Here, variables are presented as the number of occurrences (*n*) and percentage (%). There were no missing data and therefore, no imputation was performed. *p*-Values < 0.05 were considered as the threshold for the level of significance.

## Results

### Participants

A total of 57 participants, including 33 females (57.9%) and 24 males (42.2%) were included in the trial (Table 1). The participants consisted of 41 students (72.8%), 15 surgeons (26.3%), and one other participant (1.8%). The mean age was 27.5 (SD 8.2) years. Regarding handedness, 49 participants (86%) were right-handed, whereas eight participants (14%) were left-handed.

**Table 1:** Basic participant characteristics

	*n* (%)	Mean (SD)
Age [years]		27.5 (8.2)
Gender		
Female	33 (57.9)	
Male	24 (42.2)	
Handedness		
Right	49 (86)	
Left	8 (14)	
Profession		
Student	41 (71.9)	
Surgeon	15 (26.3)	
Other	1 (1.8)	
Students		
Study semester		7.3 (1.5)
Experience in laparoscopic surgery		
Yes	10 (17.5)	
No	31 (54.4)	
Physicians		
Physician's years in profession [years]		10.8 (8.4)
Physician's hierarchical status		
Resident	6 (10.5)	
Consultant	7 (12.3)	
Head of Department	2 (3.5)	
Procedures performed as first surgeon [*n*]		665.4 (928.3)
Procedures assisted [*n*]		395 (572.9)

Within the student group 10 students (17.5%) had previous experience in laparoscopic surgery. Among the participating surgeons were seven consultants (12.3%), six residents (10.5%), and two head of departments (3.5%).

### Impact of feedback modality on surgical skills

#### Peak force

In the peg transfer task, there were no significant differences between the various feedback modalities (Table 2). However, in the subgroup analysis, the student group showed a significant reduction in peak forces when using F2 (F1: 1.71 N vs. F2: 1.49 N vs. F3: 1.8 N vs. F4: 1.6 N; *p* = 0.02 for F2 vs. F3). The surgeons’ group did not show any differences in force exertion. Surgeons also showed a trend toward reduced peak forces when using any feedback modality compared to no tactile feedback, but the differences were not significant.

**Table 2:** Comparison of task time, mean non-zero force, and peak force between all feedback modalities for all participants as well as for subgroups of students and surgeons (significant *p*-values marked bold)

	All participants (*n* = 57)					Only students (*n* = 41)							Only surgeons (*n* = 15)					
	Feedback 1	Feedback 2	Feedback 3	Feedback 4	*p*-value	Feedback 1		Feedback 2		Feedback 3	Feedback 4	*p*-value	Feedback 1	Feedback 2	Feedback 3	Feedback 4	*p*-value	
	Mean (SD)	Mean (SD)	Mean (SD)	Mean (SD)		Mean (SD)		Mean (SD)		Mean (SD)	Mean (SD)		Mean (SD))	Mean (SD)	Mean (SD)	Mean (SD)		
Peg transfer																		
Time [s]	142 (4.9)	154.7 (6.2)	146.4 (5.3)	147.6 (6)	n.s	136.8 (4.3)		147.8 (6)		140.6 (4.9)	138.4 (4.7)	n.s	151.6 (11.8)	160.6 (10.4)	150.2 (8.4)	151.5 (8.3)	n.s	
Mean non-zero force [N]	0.46 (0.01)	0.45 (0.01)	0.47 (0.01)	0.47 (0.01)	n.s	0.47 (0.01)		0.45 (0.01)		0.48 (0.01)	0.48 (0.01)	**0.015**^**a**^	0.43 (0.02)	0.44 (0.02)	0.43 (0.01)	0.45 (0.01)	n.s	
Peak force [N]	1.61 (0.07)	1.45 (0.07)	1.67 (0.09)	1.61 (0.09)	n.s	1.71 (0.08)		1.49 (0.08)		1.8 (0.11)	1.6 (0.09)	**0.02**^**a**^	1.38 (0.15)	1.33 (0.14)	1.28 (0.16)	1.63 (0.22)	n.s	
Suture and knot																		
Time [s]	331.1 (18.3)	358.1 (20.4)	335.2 (19.7)	330.9 (19.9)	n.s	365.72 (20.2)		392.1 (22.1)		378.2 (21.8)	380 (21.1)	n.s	232.1 (27.8)	260.8 (36.8)	212.2 (21.4)	187.8 (16.3)		n.s
Mean non-zero force [N]	0.65 (0.01)	0.55 (0.02)	0.61 (0.01)	0.67 (0.02)	** < 0.05**^**b**^	0.67 (0.02)		0.57 (0.02)		0.62 (0.02)	0.71 (0.03)	** < 0.05**^**c**^	0.6 (0.03)	0.49 (0.03)	0.56 (0.03)	0.57 (0.04)		**0.003**^**d**^
Peak force [N]	2.4 (0.13)	2.08 (0.11)	2.11 (0.1)	2.39 (0.1)	** < 0.05**^**e**^	2.44 (0.14)		2.22 (0.14)		2.19 (0.1)	2.57 (0.12)	**0.008**^**f**^	2.3 (0.3)	1.67 (0.18)	1.89 (0.25)	1.89 (0.14)		n.s

^a^Significant *p*-values only between F2 vs. F3

^b^Significant *p*-values only between F1 vs. F2 & F3, F2 vs. F3 & F4, and F3 vs. F4

^c^Significant *p*-values only between F1 vs. F2, F2 vs. F3 & F4, and F3 vs. F4

^d^Significant *p*-values only between F1 vs. F2

^e^Significant *p*-values only between F2 vs. F4 and F3 vs. F4

^f^Significant *p*-values only between F3 vs. F4

For the laparoscopic suture and knot tasks, there was a significant distinction between the feedback modalities for the overall analysis. Again, F2 resulted in the lowest peak force application (F1: 2.4 N vs. F2: 2.08 N vs. F3: 2.11 N vs. F4: 2.39 N; *p* < 0.05 for F2 vs. F4 and F3 vs. F4). In the student group, the lowest peak forces could be observed for feedback 3 (F1: 2.44 N vs. F2: 2.22 N vs. F3: 2.19 N vs. F4: 2.57 N; *p* = 0.008 for F3 vs. F4). In the cohort of surgeons, F2 was again associated with the lowest peak force, a substantially greater peak force was applied under F1 than under peg transfer (F1: 2.3 N vs. F2: 1.67 N vs. F3: 1.89 N vs. F4: 1.89 N; *p* > 0.05); this resulted in a 22% higher averaged peak force than in the session without any feedback, yet the difference was not significant.

#### Mean non-zero force

Again, there were no significant differences in the peg transfer task regarding the mean non-zero force exerted by any of the participants (Table 2). In the subgroup analysis, only the student group showed a significant difference with F2 resulting in the lowest force application (F1: 0.47 vs. F2: 0.45 vs. F3: 0.48 vs. F4: 0.48; *p* = 0.015 for F2 vs. F3). There were no significant differences among the surgeons.

There were significant differences in the performance of the laparoscopic suture and knot task among almost all the feedback modalities. Here, feedback 4 was associated with the highest and F2 with the lowest mean non-zero forces (F1: 0.65 vs. F2: 0.55 vs. F3: 0.61 vs. F4: 0.67; *p* < 0.05 for F1 vs. F2 & F3 and F2 vs. F3 & F4 and F3 vs. F4). This dominance of F2 continued to appear in the subgroup analysis for both, the student group (F1: 0.67 vs. F2: 0.57 vs. F3: 0.62 vs. F4: 0.71; *p* < 0.05 for F1 vs. F2 and F2 vs. F3 & F4 and F3 vs. F4) and the surgeon group (F1: 0.6 vs. F2: 0.49 vs. F3: 0.56 vs. F4: 0.57; *p* = 0.003 for F1 vs. F2).

#### Task time

There were no significant differences in the task completion time between the various feedback modalities for either the peg transfer task or the laparoscopic suture and knot task. Subgroup analysis also revealed no significant differences. F2 tended to be associated with slightly longer task completion times in both tasks and in subgroup analysis (Table 2).

#### Errors

The various feedback modalities did not lead to significant differences in the occurrence of predefined errors (Table 3). Furthermore, no significant differences were apparent between the student and surgeon group.

**Table 3:** Comparison of errors between all feedback modalities for all participants as well as for subgroups of students and surgeons

	All participants (*n* = 57)					Only students (*n* = 41)					Only surgeons (*n* = 15)				
	Feedback 1	Feedback 2	Feedback 3	Feedback 4		Feedback 1	Feedback 2	Feedback 3	Feedback 4		Feedback 1	Feedback 2	Feedback 3	Feedback 4	
	*n* (%)	*n* (%)	*n* (%)	*n* (%)	*p*-value	*n* (%)	*n* (%)	*n* (%)	*n* (%)	*p*-value	*n* (%)	*n* (%)	*n* (%)	*n* (%)	*p*-value
Peg transfer															
Slip	9 (15.8)	15 (26.3)	14 (24.6)	13 (22.8)	0.499	5 (12.2)	9 (22)	8 (19.5)	7 (17.1)	0.632	4 (26.7)	6 (40)	5 (33.3)	6 (40)	0.848
Suture and knot															
Precision	25 (43.9)	27 (47.4)	23 (40.4)	22 (38.6)	0.752	20 (48.8)	20 (48.8)	16 (39)	17 (41.5)	0.699	5 (33.3)	7 (46.7)	7 (46.7)	5 (33.3)	0.753
Closure	4 (7)	2 (3.5)	3 (5.3)	3 (5.3)	0.861	0 (0)	0 (0)	1 (2.4)	1 (2.4)	0.572	4 (26.7)	2 (13.3)	2 (13.3)	2 (13.3)	0.709
Tightness	9 (15.8)	16 (28.1)	9 (15.8)	12 (21.1)	0.283	7 (17.1)	16 (39)	9 (22)	11 (26.8)	0.136	2 (13.3)	0 (0)	0 (0)	1 (6.7)	0.194

### Subjective evaluation of feedback modalities

#### Perception

The perception of F2 during peg transfer and the laparoscopic suture and knot task was rated by 85.9% and 87.8% of participants as “good” or even better, respectively (Table 4). Therefore, F2 was significantly more perceptible than F1 and F3 in both, the peg transfer task (*p* < 0.001) and the laparoscopic suture and knot task (*p* = 0.004).

**Table 4:** Comparison of all feedback modalities in terms of perception, intensity, and usefulness (significant *p*-values marked bold)

	Peg transfer				Laparoscopic suture and knot			
	Feedback 1	Feedback 2	Feedback 3		Feedback 1	Feedback 2	Feedback 3	
	*n* (%)	*n* (%)	*n* (%)	*p*-value	*n* (%)	*n* (%)	*n* (%)	*p*-value
Feedback perception								
No feedback received	15 (26.3)	0 (0)	8 (14)	** < 0.001**	6 (10.5)	0 (0)	3 (5.3)	**0.004**
Indistinct perception of feedback	5 (8.8)	3 (5.3)	8 (14)		2 (3.5)	0 (0)	2 (3.5)	
Concentration needed for perception of feedback	10 (17.5)	5 (8.8)	11 (19.3)		12 (21.1)	4 (7)	14 (24.6)	
Good perception of feedback	14 (24.6)	34 (59.6)	16 (28.1)		19 (33.3)	36 (63.2)	19 (33.3)	
Precise and very good perception of feedback	13 (22.8)	15 (26.3)	14 (24.6)		15 (26.3)	14 (24.6)	16 (28.1)	
Feedback Intensity								
No feedback received	9 (15.8)	1 (1.8)	3 (5.3)	** < 0.001**	4 (7)	1 (1.8)	0 (0)	** < 0.001**
Very weak	1 (1.8)	0 (0)	1 (1.8)		1 (1.8)	0 (0)	0 (0)	
Weak	7 (12.3)	2 (3.5)	9 (15.8)		8 (14)	1 (1.8)	8 (14)	
Well perceptible	34 (59.6)	22 (38.6)	36 (63.2)		32 (56.1)	23 (40.4)	36 (63.2)	
Strong	4 (7)	23 (40.4)	7 (12.3)		6 (10.5)	17 (29.8)	8 (14)	
Very strong	2 (3.5)	9 (15.8)	1 (1.8)		3 (5.3)	12 (21.1)	2 (3.5)	
Feedback usefulness								
No feedback received	2 (3.5)	1 (1.8)	0 (0)	0.516	1 (1.8)	0 (0)	0 (0)	0.854
Not useful	2 (3.5)	3 (5.3)	2 (3.5)		3 (5.3)	5 (8.8)	3 (5.3)	
Somewhat useful	10 (17.5)	9 (15.8)	11 (19.3)		6 (10.5)	6 (10.5)	6 (10.5)	
Useful	20 (35.1)	22 (38.6)	22 (38.6)		22 (38.6)	23 (40.4)	23 (40.4)	
Very useful	17 (29.8)	18 (31.6)	17 (29.8)		17 (29.8)	17 (29.8)	18 (31.6)	

According to the subgroup analysis, the number of students who rated F2 as at least “good” was significantly greater in the peg transfer group (F1: 61% vs. F2: 87.8% vs. F3: 63.4%; *p* = 0.025) (Table 4). Interestingly, although there were more students who rated F2 “good” or better in the laparoscopic suture and knot task (F1: 68.3% vs. F2: 90.5% vs. F3: 65.9%; *p* = 0.15), there was no significant difference. However, the number of surgeons who gave F2 the two best ratings was significantly greater for both, the peg transfer (F1: 13.3% vs. F2: 80% vs. F3: 26.7%; *p* < 0.001) and the suture and knot task (F1: 40% vs. F2: 86.7% vs. F3: 53.4%; *p* = 0.006).

#### Intensity

With 40.4% of participants reporting a “strong” and 15.8% a “very strong” intensity, significantly more participants stated that F2 had the highest intensity in the peg transfer task (*p* < 0.001). Compared to F1 and F3, F2 had a significantly greater intensity in the laparoscopic suture and knot task with 21.1% of participants reporting it to be “very strong” (*p* < 0.001).

Here, the number of students reporting a “strong” and “very strong” intensity in peg transfer (F1: 14.7% vs. F2: 61% vs. F3: 17%; *p* < 0.001) and in the suture and knot task (F1: 17.1% vs. F2: 60.9% vs. F3: 17%; *p* < 0.001) revealed the strongest feedback intensity for F2 (Table 4). During peg transfer task, significant more surgeons stated that F2 had “strong” or “very strong” intensity compared to F1 or F3 (F1: 0% vs. F2: 40% vs. F3: 6.7%; *p* < 0.001). However, in the laparoscopic suture and knot task, there was no significant difference in the ratings between the feedback modalities (*p* = 0.179).

#### Perceived usefulness

Interestingly, regarding usefulness the differences between the feedback modalities were less obvious than those for intensity and perception. Here, F1, F2, and F3 showed comparable ratings in both tasks, and the differences were not significant.

In the subgroup analysis, students did not perceive any of the feedback modalities to be more useful than the others, neither in the peg transfer task (*p* = 0.926) nor in the suture and knot task (*p* = 0.819) (Table 4). However, although more surgeons found F2 “useful” or “very useful” in both, the peg transfer (F1: 40% vs. F2: 53.3% vs. F3: 46.7%; *p* = 0.15) and the laparoscopic suture and knot task (F1: 46.6% vs. F2: 66.6% vs. F3: 53.3%; *p* = 0.105), the differences were not significant.

#### Harmony

The harmony values did not differ significantly between F1, F2, and F3 in the peg transfer task (F1: 70.3 vs. F2: 68.6 vs. F3: 70.8; *p* > 0.05) or in the laparoscopic suture and knot task (F1: 69 vs. F2: 68.9 vs. F3: 70.9; *p* > 0.05). There was also no significant difference within the subgroup analysis (Table 5).

**Table 5:** Comparison of all feedback modalities in terms of harmony and comfort

	Peg transfer				Laparoscopic suture and knot			
	Feedback 1	Feedback 2	Feedback 3		Feedback 1	Feedback 2	Feedback 3	
	Mean (SD)	Mean (SD)	Mean (SD)	*p*-value	Mean (SD)	Mean (SD)	Mean (SD)	*p*-value
Harmony of feedback [scale 1–100]	70.3 (16.3)	68.6 (18.8)	70.8 (15.7)	n.s	69 (19.1)	68.9 (18.8)	70.9 (15.4)	n.s
Comfort of feedback [scale 1–100]	69.7 (15.5)	66.4 (18.7)	69 (16.2)	n.s	69.8 (15.8)	66.8 (19)	68.9 (16.3)	n.s

#### Autotelics/comfort

All the feedback modalities showed comparable values of comfort without any significant differences in either the peg transfer (F1: 69.7 vs. F2: 66.4 vs. F3: 69; *p* > 0.05) or the laparoscopic suture and knot task (F1: 69.8 vs. F2: 66.8 vs. F3: 68.9; *p* > 0.05). In the subgroup analysis, there were no significant differences among feedback modalities in any task (Table 5).

## Discussion

The aim of this study was to investigate the influence of tactile vibration feedback on force application during laparoscopic training tasks. Focus was also placed on the differences among various tactile vibration feedback modalities regarding force application and user experience.

The present trial showed that linearly increasing feedback can significantly reduce the mean non-zero and peak forces of instrument tissue interactions compared to other tactile vibration feedback modalities and no tactile feedback.

Previous research demonstrated the advantages of haptic force feedback in laparoscopic instruments, with significantly reduced forces and improved tissue discrimination compared to conventional laparoscopic instruments [17].

Considering our data, the feedback-related improvement in force exerted was more pronounced when using a continuous tactile vibration feedback modality and in complex tasks, such as laparoscopic suture and knot. Panait et al. showed very similar results in their trial on virtual reality simulators with no differences during the peg transfer task, whereas tactile feedback significantly decreased the participants’ force exertion, mostly during more complex tasks [18]. However, the very different nature of the peg transfer and the suture and knot task could also be accountable to some extent for the observed differences in force application, since the peg transfer, for example, requires more direct contact of the instruments with the task material. According to our subgroup analysis, students benefited from continuous tactile vibration feedback in both simple (peg transfer) and complex (laparoscopic suture and knot) tasks, whereas surgeons showed a significant decrease in force exertion due to tactile vibration feedback only in terms of mean non-zero force application during complex tasks. This finding may indicate that surgical novices are more receptive to tactile vibration feedback. Additionally, surgical experience might lead to certain compensation methods to estimate appropriate force application. Similar experience-dependent compensation for the lack of haptic feedback was found for robot-assisted surgery [19, 20]. According to Meccariello et al., the significantly better performance of surgical experts was most likely due to the learning of sufficient visual compensation [19].

Previous surgical expertise, for example knowledge of the structure and properties of tissues, might also improve tissue handling. As shown by Postema et al., tactile exploration of a tissue model before performing a laparoscopic task on the same tissue model can significantly reduce the amount of force exerted [7].

One important aspect of the present trial was that the worst results in terms of force exertion, task time, or errors, even though they were not always significantly different, occurred mostly using F1 or F3 and not as expected in the context of no tactile vibration feedback. This observation indicates that tactile vibration feedback can not only improve, but may also deteriorate, laparoscopic performance.

On closer inspection, the improved force exerted when using F2 was accompanied by longer task completion time in all tasks and in subgroup analysis. Even though these differences in task completion time were not significant, it seems that the continuous vibration feedback slowed the participants down, which might eventually lead, at least in part, to favorable force application. In contrast, haptic feedback provided in robotic surgery seemed to have a positive effect on task completion time [21].

There were no significant differences among the feedback modalities regarding the occurrence of errors. These findings are in line with the results of a survey by Alleblas et al., in which surgeons rated the usefulness of haptic feedback in laparoscopy as comparatively low in terms of reduction in time or complications [1].

However, the subjective opinion might not be the most reliable source for such conclusions, as shown in our survey. Here, we observed no significant differences regarding the participants’ ratings of usefulness, harmony, and comfort among the various feedback modalities. For the perception and intensity of the tactile vibration feedback, F2 was significantly better rated than the other feedback modalities. These results might indicate that perceived usefulness does not always correlate with the objective advantage of new innovations. Nevertheless, the acceptance and actual application of a new technology could be significantly influenced by the subjective perceptions of potential users.

Although there are assumptions that the use of laparoscopic instruments with haptic feedback could improve the complication rate, e.g., for deep endometriosis, there are a lack of clinical data to support such results [22]. Therefore, clinical studies are needed to investigate the effects of laparoscopic instruments with haptic feedback to demonstrate the potential benefits for surgeons and ultimately for patients.

### Strengths and limitations

A limitation of the developed prototype is the lack of differentiation of force exerted by the left and right hands. Therefore, the vibration feedback can only represent the general force exertion and subsequently, participants were unable to track which hand exerted excessive force. This limitation might lead to a general avoidance of force application, even in situations where one hand uses a higher force deliberately. The integration of force sensors into instrument tips is still a technical challenge, yet Alleblas et al. have shown that the development of such an instrument with haptic force feedback, which theoretically could be used in clinical cases, is possible [23].

Additionally, the question remains as to what level of force should be considered surgically appropriate. Horeman et al. attempted to establish force thresholds after applying force on various animal tissues and observing when tissue damage occurred. They found that for suturing, forces below 2 N are safe for most tissues [24]. On the other hand, de Visser et al. have shown that higher tensile forces of 2.5–5 N exerted on the large intestine of pigs could be more realistic, at least in a pig model [25]. Since there are almost no further available data on force tolerance, we set the force limit for our prototype to 2 N based on the findings of Horeman et al. Nevertheless, there are organ-specific differences in force tolerance, and the data thus far published to date are based on animal experiments that may not be transferable to humans or clinical practice.

The inclusion of both surgeons and surgical novices offered a substantial advantage in this trial. With surgical novices, we were able to investigate a group of participants with high comparability after reaching proficiency in a standardized laparoscopic training course. Furthermore, the impact of experience and different education can be diminished. On the other hand, analyzing experienced surgeons provides a better reflection of clinical reality. The comparison of the two groups also allowed us to assess the influence of surgical experience to some extent. However, the number of surgeons was small and their surgical experience varied widely.

With regard to the user experience, our data initially show contradictory results. Despite the clear objective advantage of one-component feedback, the subjective survey showed no significant differences, at least in terms of usefulness. Nevertheless, it must be considered that user experience is dynamic, highly subjective and dependent on the user’s needs, prior experiences, and context [26].

## Conclusion

Continuous tactile vibration feedback with vibration pulses integrated into laparoscopic instrument handles can effectively reduce the amount of force exerted during laparoscopic surgery. Surgical novices benefitted in all tasks from this system, whereas experienced surgeons benefited only in more advanced laparoscopic tasks. This effect was most pronounced for a vibration signal linearly increasing with higher force application compared to other vibration signals and no tactile vibration feedback at all. Regarding the user experience, perceived usefulness did not correlate with actual advantage.

Therefore, this technology might be beneficial for supporting surgical novices in learning adequate tissue handling and force exertion. For future translation into clinical use, further research is needed, especially in regard to the integration of force measuring sensors within the instruments’ tips.

### Supplementary Information

Below is the link to the electronic supplementary material.

Supplementary file1 (DOCX 19 KB)
