# Supplementary material for: Can you feel the force just right? Tactile force feedback for training of minimally invasive surgery—evaluation of vibration feedback for adequate force application
Source: Surg Endosc. 2024 Jun 4;38(7):3917–28. doi: 10.1007/s00464-024-10919-3 (PMC11219423; doi:10.1007/s00464-024-10919-3)
Supplement: Supplementary file 1 — Supplementary file1 (DOCX 19 KB) [file 464_2024_10919_MOESM1_ESM.docx]

**Supplementary Material**

|  | **Feedback definition** | **Feedback pattern** |
| --- | --- | --- |
| Feedback 1 | Double vibration impulse if force application exceeds 2 N | > 2 N:   1. Pulse (intensity: 100 %: frequency: 190 Hz; duration: 50 ms) 2. Pause (duration: 120 ms) 3. Pulse (intensity: 70 %; frequency: 140 Hz; duration: 50 ms) |
| Feedback 2 | Vibration impulse increasing linearly to the force, starting at a minimal force of 0.14 N and extending over 2 N, double vibration impulse at 2 N of force application | 0.14 – 2 N:   1. Range of vibration intensity (intensity: 0 % - 100 %; frequency: 0 – 190 Hz).   > 2N:   1. 2N Pulse (intensity: 100 %: frequency: 190 Hz; duration: 50 ms) 2. Pause (duration: 120 ms) 3. Pulse (intensity: 70 %; frequency: 140 Hz; duration: 50 ms) |
| Feedback 3 | Single vibration impulse at 1.5 N and double vibration impulse if force application exceeds 2 N | = 1.5 N:   1. Pulse (intensity: 70 %; frequency: 140 Hz; duration: 50ms)   > 2 N:   1. Pulse (intensity: 100 %; frequency: 190 Hz; duration: 50ms) 2. Pause (duration: 120 ms) 3. Pulse (intensity: 70 %; frequency: 140 Hz; duration: 50 ms) |
| Feedback 4 | None | None |

Supplementary Material Table 1: Tactile feedback modalities

| **Task** | **Error Definition** |
| --- | --- |
| Peg Transfer | Lost triangle |
| Suture Precision | Suture through only one or none of the marked dots |
| Suture Approximation | Both sides of the suture dummy do approximate but do not touch or do not approximate at all |
| Knot tightness | Knot loosens by slight manipulation or visibly loose knot |
| Supplementary Material Table 2: Error definitions | |

| **Dimension** | **Survey question** | **Response options** |
| --- | --- | --- |
| Perception | How was the feedback perceived? | 1 – I have not felt any feedback  2 – I could only vaguely perceive the feedback  3 – I had to concentrate on the feedback in order to perceive it  4 – I was able to perceive the feedback well  5 – The feedback was precise and very well perceptible |
| Intensity | What did the feedback feel like? | 1 – Very weak  2 – Weak  3 – Well perceptible  4 – Strong  5 – Very strong |
| Usefulness | How useful would you consider this feedback in fulfilling the tasks? | 1 – Not useful  2 – Somewhat useful  3 – Useful  4 – Very useful |
| Harmony | How well does the feedback fit into the task situation? | 0 – No feedback  1 – Very bad  100 – Very good |
| Autotelics/Comfort | How does the feedback feel? | 0 – No feedback  1 – Uncomfortable  100 – Comfortable |
| Supplementary Material Table 3: Survey for user experience and subjective evaluation of feedback modalities | | |
